# A Suggestion about Cocain

**Published:** 1896-01

**Authors:** G. E. Hunt

**Affiliations:** Indianapolis


### A Suggestion About Cocain.

BY G. E. HUNT, D.D.S., INDIANAPOLIS.

Abstract of paper read at American Dental Association, August, 1895.

A survey of the situation leads me to think that the percent. solution is largely responsible for the various ill effects observed. The great majority of dentists, I believe, get their two, three or four percent. solutions put up by a druggist and their ideas of how many grains of cocain are required to make a four percent. solution are often hazy.

In the majority of cases an eighth or a quarter of a grain will accomplish the desired result, while the maximum dose, one-half grain, would be attended with, at least, temporary ill effects. I would suggest that the percent. solution be entirely ignored in using cocain, *per se*, and that the intended dose be dissolved in an indefinite, convenient quantity of water and the entire amount exhibited.

Following this plan of procedure will impress *dosage* on the operator in a manner that no other method can succeed in doing. Another, and a potent argument in favor of this mode, is the fact that every solution used is a fresh one. The rapid deterioration of cocain solutions renders the degree of effects produced from twenty-four or forty-eight hour solutions extremely problematical. By dissolving the cocain as it is needed, and only a short time is necessary to accomplish this, the full effects of the drug are assured.

In order to use cocain in this way it becomes necessary to have a pair of balances for the accurate measurement of the drug. These balances will, however, be found useful in various ways and should be part of the equipment of every well-conducted dental office, whether cocain is used or not.

